# The Early Experience of Synchrony in Ultra-Hypofractionation for Prostate Cancer: A Case Series

**DOI:** 10.7759/cureus.84323

**Published:** 2025-05-18

**Authors:** Piyapasara Toapichattrakul, Pooriwat Muangwong, Anupong Kongsa, Wannapha Nobnop, Anirut Watcharawipha, Ekkasit Tharavichitkul

**Affiliations:** 1 Division of Radiation Oncology, Department of Radiology, Faculty of Medicine, Chiang Mai University, Chiang Mai, THA

**Keywords:** case, experience, prostate cancer, synchrony, ultra-hypofractionation

## Abstract

Ultra-hypofractionated radiotherapy has emerged as an effective treatment for localized prostate cancer, offering comparable oncologic outcomes to conventional fractionation while significantly reducing treatment duration. However, the delivery of high doses per fraction demands exceptional precision to minimize toxicity risks, particularly in the context of intrafractional prostate motion. Real-time tracking systems, such as Synchrony (Accuray, Sunnyvale, CA, USA), aim to address this challenge by continuously monitoring and correcting for target displacement during beam delivery. This study evaluates the technical feasibility and early clinical outcomes of ultra-hypofractionated radiotherapy (36.25 Gy in five fractions) delivered with Synchrony real-time tracking, with specific analysis of prostate specific antigen (PSA) kinetics, treatment delivery parameters, and safety.

Five consecutive patients received fiducial-based radiotherapy with continuous motion tracking. PSA levels were monitored during the first two months post-treatment. Treatment efficiency metrics (including beam interruptions and motion events) and acute toxicity (Common Terminology Criteria for Adverse Events (CTCAE) v5.0) were prospectively recorded. A rapid biochemical response was observed, with a mean PSA decline rate of -4.27 ng/mL/month. System performance demonstrated stable rigid-body tracking with a median deviation of 1.04 mm (IQR: 0.74 to 1.39 mm), and clinically acceptable posterior displacement in the Z-axis with a median of -1.54 mm (IQR: -2.80 to -0.38 mm). The composite 3D target offset had a median of 2.52 mm (IQR: 1.53 to 3.30 mm). Automatic pauses occurred when motion exceeded safety thresholds, increasing mean treatment duration by 13.9% (actual: 586.5 seconds vs. planned: 514.9 seconds). Despite systematic posterior tracking offsets, toxicity was minimal (one grade 1 GI event).

Synchrony tracking enabled precise ultra-hypofractionated delivery with submillimeter accuracy and minimal toxicity. The modest increase in treatment time is a justifiable trade-off for real-time motion adaptation. These early results support further investigation into Synchrony-optimized prostate ultra-hypofractionated radiotherapy.

## Introduction

Ultra-hypofractionated radiotherapy is increasingly used for prostate cancer, aiming to deliver highly conformal doses in fewer fractions (more than 6 Gy per fraction). Randomized trials have demonstrated the non-inferior biochemical outcome and toxicity compared to dose-escalated conventional fractionation [1-7].

However, the precision required for safe dose delivery in ultra-hypofractionated radiotherapy faces a significant challenge: intrafraction prostate motion. Studies have shown that prostate displacement exceeding 5 mm occurs in more than 75% of treatment fractions, primarily due to bladder filling and bowel movement [8,9]. Such motion patterns risk both target underdosing and increased dose to adjacent organs at risk, particularly concerning when delivering these higher doses per fraction.

Several motion management systems exist for prostate ultra-hypofractionation, each with distinct technical approaches. These include magnetic resonance (MR)-guided linear accelerators (MR-LINAC), electromagnetic tracking systems (Calypso; Varian Medical Systems, Palo Alto, CA, USA), and robotic arm-mounted LINACs (CyberKnife; Accuray, Sunnyvale, CA, USA). MR-guided systems, in particular, allow direct visualization of the prostate and can detect both translational and rotational shifts, contributing to a more detailed understanding of prostate motion during treatment [10]. However, clinical implementation is often constrained by platform availability, with many centers limited to conventional LINAC-based solutions. The Synchrony system on the Radixact platform (Accuray) offers a fiducial-based tracking alternative for facilities without access to these specialized technologies. While this technology theoretically improves treatment accuracy, its practical implementation in ultra-fractionation radiotherapy remains limited [11,12].

This case series investigates the early real-world performance of Synchrony motion tracking during ultra-hypofractionated prostate radiotherapy, with particular focus on motion and characterizing treatment interruptions and their operational impact. Through comprehensive analysis of delivery parameters and motion compensation events, this study aims to evaluate the system’s clinical feasibility and identify potential optimization opportunities for prostate radiotherapy workflows.

## Case presentation

Five patients with localized prostate cancer were enrolled in this study. All suitable candidates for ultra-hypofractionated radiotherapy and were treated at Chiang Mai University Hospital.

Radiotherapy planning and preparation

Prior to CT simulation, each patient underwent transperineal implantation of three gold fiducial markers under transrectal ultrasound guidance and local anesthesia. This procedure was performed by a specialized urologist to ensure accurate placement for optimal target tracking. The image set of a Computed Tomography (CT) with 1 mm of slice thickness was acquired from the Computed Tomography simulator (SOMATOM Definition AS, Siemens Healthineers, Erlangen, Germany).

**Figure 1:**
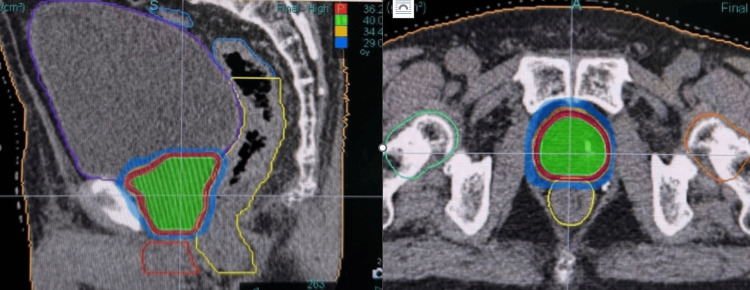
Ultra-hypofractionation in prostate cancer – Dose distribution of case #3

For treatment planning, the clinical target volume (CTV) was defined as the entire prostate gland. To account for setup uncertainties, a uniform 3-mm isotropic expansion was applied to the CTV to generate the planning target volume (PTV). Organs at risk (OARs), including the bladder, rectum, and urethra, were contoured, and appropriate dose constraints were applied to minimize toxicity. For planning we used the VOLO ultra (Accuray, Inc., Madison, WI, USA) for dose calculation system, with a prescription of 36.25 Gy in five fractions of 7.25 Gy to the prostate PTV. OAR constraints were defined for the bladder, rectum, penile bulb, and femoral heads. Treatment plans were evaluated using adapted criteria from the PACE-B trial with strict dosimetric criteria as follows (Table 1, Figure 1) [3].

**Table 1:** Dose-volume constraints for ultra-hypofractionated radiotherapy. From [3] Abbreviation: V=volume; PTV= planning target volume; cc= cubic centimeter

Structure	Dose Constraint	
PTV	V_42.8Gy_	< 2%
	V_36.25Gy_	≥ 95%
	V_34.4Gy_	≥ 98%
Bladder	V_37Gy_	< 5 cc.
	V_18Gy_	< 40%
Rectum	V_36Gy_	< 1 cc.
	V_18Gy_	< 50%
Right and Left femoral head	V_14.5Gy_	< 5%

**Figure 2:**
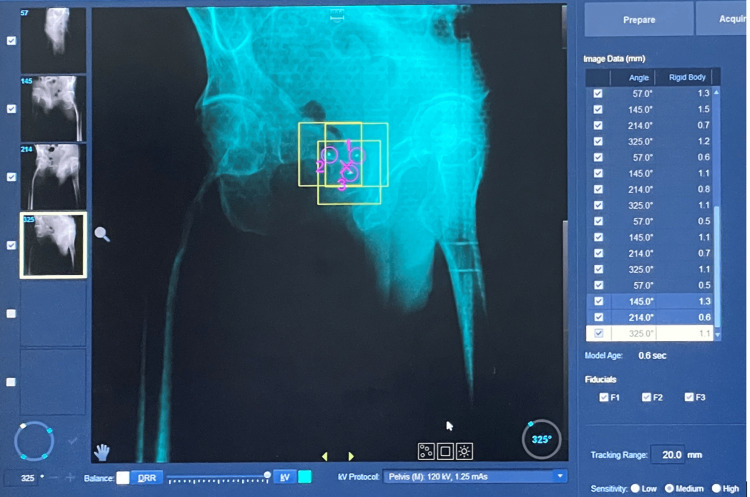
Synchrony platform in monitor

Treatment was delivered using the Synchrony system on the Radixact platform (Accuray, Inc.) with daily helical kV CT (ClearRT) for initial patient setup via fiducial marker matching. Intrafractional motion was monitored by acquiring 2D kV radiographic images (six times per gantry rotation), enabling real-time fiducial tracking. The Synchrony stereotactic body radiation therapy (SBRT) delivery adhered to predefined motion thresholds: potential difference (3D distance error): ≤ 2 mm, target offset (predicted displacement from planning CT): ≤30 mm, and rigid body constraint (fiducial pair distance deviation): ≤ 1.5 mm. If any threshold was exceeded, radiation delivery was automatically paused, and a corrective intrafraction kV CT was acquired at predefined gantry angles (35°, 145°, 215°, 325°) to reposition the target. The Synchrony tracking system (Figure 2) enabled continuous real-time monitoring during treatment delivery. Comprehensive dosimetric and motion parameters were recorded for quality assurance, including PTV coverage, OAR doses, fiducial marker displacement, and the frequency of treatment interruptions per fraction.

Results

The study consisted of five patients with localized prostate cancer who underwent ultra-hypofractionated radiotherapy. Detailed patient characteristics, including demographics, disease staging, and treatment parameters, are presented in Table 2. The mean CTV was 42.65 cc (range: 25.08-66.32 cc.) (Table 3). All patients met target coverage constraints. While most OAR doses remained within protocol limits, two cases had higher than constraints: Case 3 exceeded rectal V36Gy (1.1 cc vs. <1 cc constraint) and bladder V37Gy (6.7 cc vs. <5 cc), while Case 4 showed elevated bladder V37Gy (6.5 cc). All other OAR parameters across the cohort were maintained below constraints.

**Table 2:** Baseline characteristics and early treatment outcomes of prostate cancer patients treated with ultra-hypofractionated radiotherapy Abbreviations: GS=Gleason score; PSA=prostate specific antigen; T=tumor; N=node; M=metastasis; E=Enantone; PTV=planning target volume; CTV=clinical target volume; Tx=treatment; S=surgical castration; Abi=abiraterone acetate; ADT=androgen deprivation therapy; RT=radiotherapy

Case	Age	PSA before RT	GS	T stage	N stage	M stage	Risk group	Type of ADT	Medications	PSA 2-mo post Tx	PSA Change/month
1	77	7	3+4	2a	0	0	Favorable intermediate	E	None	1.94	-2.53
2	63	4.2	3+4	2a	0	0	Favorable intermediate	None	None	0.04	-2.08
3	70	8.5	3+4	2a	0	0	Favorable intermediate	E	None	0.07	-4.21
4	73	14.5	3+3	2	0	1b	N/A	S	Abi	0.061	-7.21
5	77	11	3+4	2a	0	0	Unfavorable intermediate	E	None	0.4	-5.3

**Table 3:** Dosimetric parameters for target volumes and organs at risk Abbreviations: CTV=clinical target volume; PTV=Planning target volume; B=bladder; R=rectum; cc=cubic centimeter

Case	Vol of PTV	Vol of CTV	V_34.4_PTV (%)	V_36.25_PTV (%)	V_42.8_PTV (%)	V_37_B (cc.)	D_50_B (%)	V_36_R (cc.)	V_18_R (%)
1	61.52cc	25.08cc	99.9%	97.3%	0%	0.67	39.4	0.82	47.1
2	122.38cc	66.32cc	100%	96.6%	0%	1.51	27.8	0.04	32.6
3	83.35cc	42.14cc	99.9%	95.6%	0%	7.63	29.6	2.67	32
4	69.44cc	45.95cc	100%	98.1%	0%	5.81	8.4	0.8	36.4
5	58.55cc	33.72cc	99.9%	95.8%	0%	3.88	12	0.96	45

Treatment delivery

The Synchrony tracking data from five patients demonstrates systematic motion patterns with clinical relevance. The system showed acceptable overall tracking accuracy, as shown in Table 4. Notably, there was a pronounced posterior shift in the Z-axis with a median displacement of -1.54 mm (IQR: -2.80 to -0.38 mm) and a substantial composite 3D variability with a median of 2.52 mm (IQR: 1.53 to 3.30 mm). The overall target offset is calculated by sqrt(𝑋^2^ + 𝑌^2^ + 𝑍^2^).

**Table 4:** Synchrony respiratory motion tracking performance across five patients reported in millimeter (mm) Note: Potential Differentiation = A statistical estimate of the predicted 3D positional error when the Synchrony model forecasts future target positions. This metric represents the system's anticipated tracking accuracy during treatment delivery. Target Offset: The measured discrepancy between the planned target position (from treatment planning) and the actual target position observed during treatment delivery. This real-time difference reflects the system's tracking performance. Rigid Body: The maximum observed deviation in inter-fiducial distances, comparing the planned 3D fiducial configuration to their actual 2D projected positions during treatment. This metric quantifies fiducial marker constellation deformation.

Parameter	Median (mm)	Interquatile range (mm)
Potential Differentiation	0.75	0.46 to 1.34
Rigid Body	1.04	0.74 to 1.39
Target Offset X	0.96	-0.72 to 0.66
Target Offset Y	-0.26	-1.15 to 0.35
Target Offset Z	-1.54	-2.80 to -0.38
Overall Target Offset (3D)	2.52	1.53 to 3.30

The mean value of planning beam-on time and actual beam-on time were 514.9 and 586.5 seconds, respectively. The average percentage of additional time was 13.9%. For the details of pausing, all pauses during treatment of these patients are shown in Table 5.

**Table 5:** Treatment delivery characteristics: Planned beam-on times (BOT), number(s) of treatment interruption, and time efficiency across fractions. Abbreviations: Tx=treatment; AVG=average; s=second

No	planned BOT (s)	1st		2nd		3rd		4th		5th		Avg Tx time from 5 Fx (s)	% incresing time compare to planned BOT
		Tx time (s)	Number of Interruption (#)	Tx time (s)	Number of Interruption (#)	Tx time (s)	Number of Interruption (#)	Tx time (s)	Number of Interruption (#)	Tx time (s)	Number of Interruption (#)		
1	529.7	1094	14	555	0	555	0	559	0	641	2	680.8	28.53%
2	598.2	633	0	628	0	656	3	623	0	631	0	634.2	6.02%
3	488.3	523	0	523	0	562	1	670	4	548	1	565.2	15.75%
4	448.4	481	0	473	0	481	0	473	0	503	1	482.2	7.54%
5	509.7	534	0	585	1	612	2	578	1	542	0	570.2	11.87%

The treatment was well-tolerated, with only one patient (20%) developing acute grade 1 gastrointestinal toxicity (diarrhea) according to Common Terminology Criteria for Adverse Events (CTCAE) v5.0 criteria. This resolved completely with supportive care.

## Discussion

This case series demonstrates the successful implementation of ultra-hypofractionated prostate radiotherapy using the Synchrony real-time motion tracking system, with acceptable dosimetric outcomes and no acute high-grade toxicities. Our findings contribute to the growing evidence supporting ultra-hypofractionation RT for localized prostate cancer, while highlighting the unique advantages of continuous intrafraction tracking compared to daily image-guided radiotherapy [1-7].

In our initial experience with ultra-hypofractionated radiotherapy (36.25 Gy in five fractions), we observed a remarkably rapid PSA decline averaging -4.27 ng/mL per month. This substantially exceeds the previously reported decline rates of -0.47 to -0.09 ng/mL per month in the first year post-treatment, reinforcing existing evidence that ultra-hypofractionation induces faster biochemical response kinetics compared to conventional fractionation regimens [13]. However, interpretation requires caution: 80% of our cohort received concurrent ADT (castration: 4/5; abiraterone: 1/5), which likely augmented the early PSA dynamics. While these results align with the hypothesis that ultra-hypofractionation may enhance tumor control, the confounding effects of systemic therapy preclude definitive attribution of biochemical response solely to radiation.

For the toxicity report, with a median follow-up of two months, revealed only one case of acute Grade 1 GI/GU toxicity - a notably lower incidence compared to the 53-57% rate reported in the PACE-B trial at 12 weeks [3-6]. This discrepancy may stem from several factors, including the use of real-time tumor tracking via Synchrony, which enhances precision in dose delivery, as well as our more conservative CTV-to-PTV expansion (3 mm vs. PACE-B’s 5-7 mm). Additionally, our prescribed dose did not include escalation to 40 Gy for the CTV, unlike PACE-B, where select patients received higher doses to dominant lesions. However, the shorter follow-up duration in our study limits the assessment of late toxicities, which typically emerge beyond six months. While these early results suggest that advanced motion management and tighter margins may reduce acute toxicity, longer-term data will be essential to fully evaluate the safety profile of our approach compared to established protocols.

The Synchrony system demonstrated clinically excellent motion tracking performance, with all directional displacements (X, Y, and Z axes) maintaining means and standard deviations below 5 mm - similar to the previously reported values for alternative tracking systems [9,14]. Notably, the observed posterior moved showed in the Z-axis represented the most significant directional variation, though still within clinically acceptable thresholds [15,16]. These motion patterns triggered automatic safety pauses primarily during initial fractions, with setup variations being the most frequent cause. The system's efficient correction mechanism limited the total treatment time increase to under 15% compared to planned duration, while ensuring continuous positional accuracy. Though these findings are based on a five-patient cohort, they suggest Synchrony offers superior motion management for prostate SBRT compared to existing technologies, warranting further investigation in larger studies.

## Conclusions

Synchrony successfully enhances ultra-hypofractionated prostate radiotherapy by providing dynamic motion management throughout treatment delivery. The system's automatic correction mechanism maintains treatment precision through controlled pauses when needed, while its consistent tracking performance ensures reliable target localization. This operational approach supports both therapeutic effectiveness - as evidenced by rapid biochemical response - and an excellent safety profile, confirming its value in optimizing prostate ultra-hypofractionation delivery. However, as these findings are based on a small cohort of five patients, further studies with larger sample sizes are needed to validate these results.
